# Complete chloroplast genome of *Synsepalum dulcificum* D.: a magical plant that modifies sour flavors to sweet

**DOI:** 10.1080/23802359.2020.1798299

**Published:** 2020-07-30

**Authors:** Ying-feng Niu, Shu-bang Ni, Jin Liu

**Affiliations:** Yunnan Institute of Tropical Crops, Xishuangbanna, China

**Keywords:** *Synsepalum dulcificum* D., chloroplast genome, phylogenetic relationship

## Abstract

*Synsepalum dulcificum* D. belongs to the Sapotaceae family, which is an evergreen shrub native to tropical West Africa. It is a kind of magical plant that has the unique characteristic of modifying sour flavors to sweet. In this study, the chloroplast genome of *S. dulcificum* was sequenced, assembled, and annotated firstly. Chloroplast genome size of *S. dulcificum* is 158,463 bp, the circular chloroplast genome consists of four regions: a large single-copy region of 88,256 bp, two inverted repeat regions of 25,958 bp, and a small single-copy region of 18,669 bp, with the GC content of 36.87%. A total of 133 genes were annotated in the *S. dulcificum* chloroplast genome, of which 88 were protein-coding genes (PCGs), 37 were transfer RNA (tRNA) genes, and eight were ribosomal RNA (rRNA) genes. Phylogenetic analysis showed that *Pouteria campechiana* was most closely related to *S. dulcificum*. The study provides important genomic data for further utilization and breeding of *S. dulcificum*.

*Synsepalum dulcificum* D. belongs to the Sapotaceae family (Shi et al. 2016), commonly known as miracle berry, miracle fruit, magic fruit, miraculous or asaba (Coronel et al. 2009; Obafemi et al. 2017), which is an evergreen shrub native to tropical West Africa (Wang et al. 2011). It is a kind of magical plant that has the unique characteristic of modifying sour flavors to sweet (Du et al. 2014). It is also a well-known natural source of ‘miraculin,’ a sweetening glycoprotein (Tchokponhoué et al. 2019). All plant parts of *S. dulcificum* are of medicinal importance. The berry fruit and leaf contains many nutrients along with many beneficial characteristics (Nkwocha et al. 2014; Njoku et al. 2015). It has the ability to improve insulin sensitivity, and antioxidant and anticancer abilities (Swamy et al. 2014). Thus, it may be used as an adjuvant for treating diabetic patients with insulin resistance (Chen et al. 2006; Obafemi et al. 2017). The seed oil of *S. dulcificum* is also a rare and exotic fruit oil (Guney and Nawar 1977).

As a valuable plant species, currently, *S. dulcificum* is utilized in cosmetics and food. Besides, it is also used extensively in the pharmaceutical industry (Achigan-Dako et al. 2015). Surprisingly, there are few studies on the genomics of *S. dulcificum*, focusing on the nuclear, mitochondrial and chloroplast genome, which severely limits its utilization and breeding. In this study, the chloroplast genome of *S. dulcificum* was sequenced, assembled, and annotated firstly.

Young leaves of *S. dulcificum* were collected from the Xishuangbanna Tropical Flowers and Plants Garden (100.789433 E, 22.015939 N) and frozen in liquid nitrogen. The genomic DNA was extracted using the Dneasy Plant Mini Kit (Qiagen) and then stored in the ultra-low temperature specimen library at YITC (specimen accession number: YITC-2020-FZ-S-013). DNA was sequenced using the Illumina Hiseq 2500 Platform (Illumina, San Diego, CA). The complete chloroplast genome was assembled with CLC Genomics Workbench v3.6 (http://www.clcbio.com) and annotated with Dual Organelle GenoMe Annotator (DOGMA; Wyman et al. 2004). The chloroplast genome was submitted to GenBank (http://www.ncbi.nlm.nih.gov/) under the accession number MT723946, and a physical map was generated by OGDRAW (http://ogdraw.mpimp-golm.mpg.de/) (Lohse et al. 2013).

Chloroplast genome size of *S. dulcificum* is 158,463 bp. Like other plants, the circular chloroplast genome consists of four regions: a large single-copy region of 88,256 bp, two inverted repeat regions of 25,958 bp, and a small single-copy region of 18,669 bp. The composition of the four bases is 49,395 bp of A, 50,640 bp of T, 28,594 bp of G, and 29,834 bp of C, with the GC content of 36.87%. There are 88 protein-coding genes (PCGs), eight ribosomal RNA (rRNA) genes, and 37 transfer RNA (tRNA) genes, with a total number of 133 genes that were annotated in the *S. dulcificum* chloroplast genome.

A maximum-likelihood (ML) tree based on the sequences of protein-coding genes of *S. dulcificum* and other 23 plant species was constructed to verify its phylogenetic relationship. The names of 70 genes common to all species which were used to construct the phylogenetic tree are as follows: *Ndhb*, *Rps12*, *Rps11*, *Petn*, *Ndhf*, *Rps16*, *Ndhd*, *Rps14*, *Ndhj*, *Ndhk*, *Rps18*, *Rpl2*, *Ycf3*, *Rps7*, *Psab*, *Rps4*, *Rps3*, *Rps2*, *Rpl22*, *Rps8*, *Petd*, *Petg*, *Peta*, *Rps15*, *Petb*, *Rpoc1*, *Petl*, *Ycf4*, *Rpoc2*, *Ycf2*, *Rpl20*, *Rpl23*, *Rpob*, *Atpi*, *Atph*, *Atpb*, *Atpa*, *Ndhg*, *Atpf*, *Atpe*, *Psbd*, *Psba*, *Psaj*, *Psbc*, *Psbb*, *Psbm*, *Ndhc*, *Psbn*, *Psbi*, *Psbh*, *Psbk*, *Clpp*, *Psbt*, *Rpl16*, *Ndhh*, *Rpoa*, *Rpl14*, *Rpl36*, *Rpl33*, *Infa*, *Psaa*, *Ndhi*, *Psai*, *Ndha*, *Rbcl*, *Ndhe*, *Ccsa*, *Matk*, *Cema,* and *Psac.* Multiple sequence alignment was done with MAFFT (Katoh and Standley 2013) and ML analysis was done with MEGA7.0 (Kumar et al. 2016). The 24 plant species included in the phylogenetic tree have been derived from the following families: Theaceae (10 species), Symplocaceae (two species), Sapotaceae (five species) and Pentaphylacaceae (six species), and *Hydrangea petiolaris* which belongs to Hydrangeaceae family and was used as the out group. The result showed that *S. dulcificum* was most closely related to *Pouteria campechiana* (Figure 1). The study provides important genomic data for further utilization and breeding of *S. dulcificum*.

## Data Availability

The data that support the findings of this study are openly available in GenBank at https://www.ncbi.nlm.nih.gov/, reference number MT723946. Maximum-likelihood tree based on the sequences of protein-coding genes of 24 plant species, include 10 species of Theaceae family, two species of Symplocaceae family, five species of Sapotaceae family, and six species of Pentaphylacaceae, and *Hydrangea petiolaris* which belongs to Hydrangeaceae family was used as the outgroup. The species and chloroplast genome accession numbers for the phylogenetic tree construction are *Schima superba* (KY406788)*, Schima sericans* (KY406779), *Gordonia lasianthus* (KY406790), *Gordonia brandegeei* (KY406761), *Camellia szechuanensis* (KY406778), *Camellia granthamiana* (NC_038181), *Stewartia sinii* (NC_041470), *Stewartia villosa* (NC_041469), *Hartia laotica* (NC_041509), *Stewartia serrata* (NC_041467), *Symplocos paniculata* (MG719832), *Symplocos costaricana* (NC_035708), *Argania spinosa* (MK533159), *Sideroxylon wightianum* (NC_041130), *Manilkara zapota* (MN295595), *Synsepalum dulcificum* D. (MT723946), *Pouteria campechiana* (KX426215), *Adinandra millettii* (MF179492), *Adinandra angustifolia* (MF179491), *Euryodendron excelsum* (NC_039178), *Ternstroemia gymnanthera* (MF179490), *Anneslea fragrans* (MF179497), *Pentaphylax euryoides* (MF179498), and *Hydrangea petiolaris* (KY412466).
